# Neuroendoscopic Removal of Acute Subdural Hematoma with Contusion: Advantages for Elderly Patients

**DOI:** 10.1155/2016/2056190

**Published:** 2016-02-15

**Authors:** Ryota Tamura, Yoshiaki Kuroshima, Yoshiki Nakamura

**Affiliations:** Department of Neurosurgery, Tokyo Medical Center, 2-5-1 Higashigaoka, Meguro-ku, Tokyo 152-8902, Japan

## Abstract

*Background*. Large craniotomy for acute subdural hematoma is sometimes too invasive. We report good outcomes for two cases of neuroendoscopic evacuation of hematoma and contusion by 1 burr hole surgery.* Case Presentation.* Both patients arrived by ambulance at our hospital with disturbed consciousness after falling. Case 1 was an 81-year-old man who took antiplatelet drugs for brain infarction. Case 2 was a 73-year-old alcoholic woman. CT scanning showed acute subdural hematoma and frontal contusion in both cases. In the acute stage, glycerol was administered to reduce edema; CTs after 48 and 72 hours showed an increase of subdural hematoma and massive contusion of the frontal lobe. Disturbed consciousness steadily deteriorated. The subdural hematoma and contusion were removed as soon as possible by neuroendoscopy under local anesthesia, because neither patient was a good candidate for large craniotomy considering age and past history. 40%~70% of the hematoma was removed, and the consciousness level improved.* Conclusion*. Neuroendoscopic removal of acute subdural hematoma and contusion has advantages and disadvantages. For patients with underlying medical issues or other risk factors, it is likely to be effective.

## 1. Introduction

Hematoma evacuation by large craniotomy is the standard treatment for acute subdural hematoma (ASDH) with brainstem compression. Craniotomy in general is known to impose a significant burden on patients due to the large amount of bleeding, large skin incision, and long operation time. It also requires general anesthesia, which adds to the burden. Therefore, there are many patients who are considered unsuitable for large craniotomy, because of antiplatelet or anticoagulation drugs, hepatic cirrhosis, or older age. In contrast, neuroendoscopy hematoma evacuation is a minimally invasive procedure, requiring only a 4 cm skin incision and 1 burr hole. It can be performed under local anesthesia and mild sedation. Here we report good outcomes for two patients who underwent neuroendoscopic procedure for hematoma and contusion evacuation.

To our knowledge, there are no previous reports of this procedure performed for ASDH with concomitant contusion.

## 2. Case Presentations

### 2.1. Case 1

**Figure 1:**
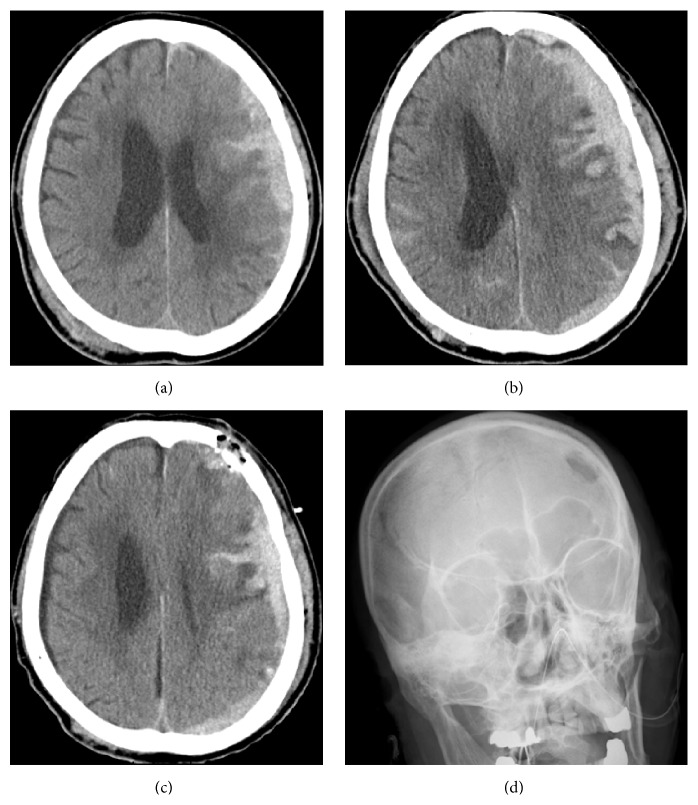
(a) Axial plain CT scan at the time of admission shows left acute subdural hematoma and bilateral frontal contusion with thickness of 14 mm and midline shift of 8 mm. There is a bruised area in the right parietal region without bone fracture. (b) Axial plain CT scan 72 hours after admission shows worsened acute subdural hematoma with thickness of 16 mm and midline shift of 9 mm. Massive contusion of the left frontal lobe has occurred. (c) Radiographic frontal view shows the location of the burr hole 4 cm above the left eyebrow. (d) Axial plain CT scan after surgery shows reduced hematoma. Midline shift had improved to 4 mm. There is a small amount of air in the subdural space. Burr hole is covered by bone powders.

An 81-year-old man presented to our hospital by ambulance with disturbed consciousness after falling. He was taking the antiplatelet drug cilostazol for brain infarction. The admission Glasgow Coma Scale (GCS) score was 13 (E4V4M5). Computed tomography (CT) scanning revealed left ASDH and bilateral frontal contusion with a thickness of 14 mm and a midline shift (MLS) of 8 mm (Figure 1(a)). Our initial plan was to give conservative treatment. In the acute stage, tranexamic acid (2000 mg) was administered to staunch the bleeding. But CT 24 hours later revealed worsening (thickness 16 mm, MLS 8 mm). Contusion in the left frontal lobe became especially apparent. Glycerol (1600 mL/day) was administered to reduce edema, but the 72-hour CT showed massive contusion of the left frontal lobe and the MLS had increased to 9 mm (Figure 1(b)). The GCS score deteriorated steadily to E2V2M4. At this point, decision to perform surgery was made. As for the method of surgery, neuroendoscopy under local anesthesia and mild sedation was chosen, since the patient was not a good candidate for large craniotomy considering his age and the use of antiplatelet drug. A 4 cm skin incision was made along the shriveled skin of the left forehead 4 cm above the eyebrow in the hairline, and 1 burr hole was made using a hand drill. We then formed the hole into an earthenware mortar shape and made a cross-dural incision to expose the brain surface. A 10 mm diameter sheath (Neuroport, Olympus Corp.) was inserted into the brain and the contusion was removed first using a rigid scope (0°, 2.7 mm). We then guided the Neuroport to the subdural space and removed the subdural hematoma as completely as possible.

In total, 40% of the hematoma was removed, and the MLS was improved to 4 mm after the procedure (Figures 1(c) and 1(d)). Tranexamic acid (250 mg) was administered to prevent postoperative oozing.

The consciousness level started to improve right after the operation and eventually improved to E4V3M5 20 days after operation. The patient could eat without assistance by this time. Kampo (goreisan) was prescribed to prevent chronic subdural hematoma.

### 2.2. Case 2

**Figure 2:**
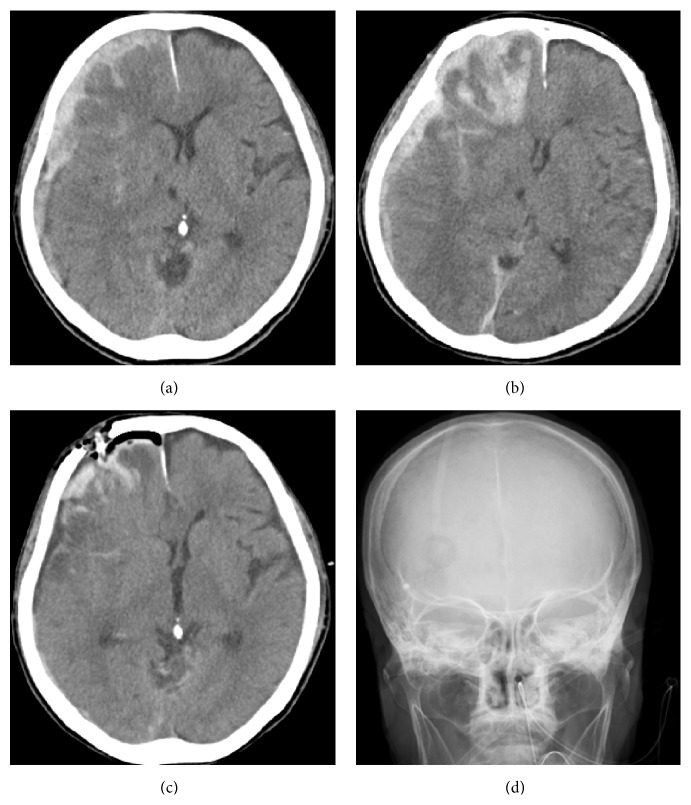
(a) Axial plain CT scan at the time of admission shows right acute subdural hematoma and right frontal and temporal contusion with thickness of 10 mm and midline shift of 6 mm. There is a bruised area in the left temporal region without bone fracture. (b) Axial plain CT scan 48 hours after admission showed massive contusion and uncal herniation. The midline shift has worsened to 9 mm. (c) Most hematoma was removed and midline shift was completely resolved. The massive contusion in the right frontal lobe was reduced. The information drain was inserted into the subdural space. (d) Radiographic frontal view shows location of the burr hole 3 cm above the right eyebrow.

A 73-year-old woman presented to our hospital by ambulance with disturbed consciousness after drinking alcohol and falling. Her past medical history was diabetes and alcohol abuse. Her admission GCS score was 14 (E4V4M6). CT scanning showed right ASDH and right frontal and temporal contusion with a thickness of 10 mm and an MLS of 6 mm (Figure 2(a)). Our initial plan was to give conservative treatment. In the acute stage, tranexamic acid (2000 mg) and glycerol were administered, as with Case 1. But the CT after 48 hours showed edema around the contusion and uncal herniation with anisocoria (Figure 2(b)). Since the massive contusion in the frontal lobe exerted a mass effect, removal of contusion was considered.

We removed the subdural hematoma and contusion to the furthest extent possible by neuroendoscopy under local anesthesia and mild sedation, as with Case 1. We placed a 4 cm incision on the forehead outside of the hairline in order to remove the massive contusion together with the subdural hematoma. Considering cosmetic outcomes, incision was made parallel to the wrinkle lines and 5-0 nylon suture was used for skin closure. In total, 70% of the hematoma was removed, and the MLS improved completely (Figures 2(c) and 2(d)). Tranexamic acid (250 mg) was administered to prevent postoperative oozing.

The consciousness level started to improve right after the operation and eventually improved to E4V4M6 27 days after the operation. The skin incision was hardly noticeable after suture removal.

## 3. Discussion

### 3.1. Indications

Surgical treatment often considered for ASDH is large craniotomy hematoma evacuation. However, craniotomy in general imposes a significant burden on patients due to the large amount of bleeding, large question mark skin incision, and long operation time under general anesthesia. Therefore, the procedure may be inadvisable for patients with medical conditions such as liver cirrhosis, older age, and the use of antiplatelet/anticoagulation drugs.

In contrast, neuroendoscopic surgery is a minimally invasive technique that can be performed under local anesthesia and therefore can be applied to patients who may not endure craniotomy. For example, it is considered suitable for elderly patients with complications such as heart failure. In such cases, reduction of antiedema drugs will become possible after the surgery, thus preventing the exacerbation of heart failure. However, there are few reports of neuroendoscopic surgery on ASDH. Although there are increasing reports on neuroendoscopic removal of chronic subdural hematoma (CSDH), removal of ASDH is considered difficult because of its gelatinous nature as opposed to the serous nature of CSDH [1–3]. Our literature research revealed only one report of neuroendoscopic surgery for pure ASDH. It was a case of ASDH (width 15 mm, MLS 14 mm) of an 84-year-old woman with GCS of E1VTM6 who fell a week before the surgery. Hematoma was removed through 2 perforating burr holes at the front and back of the convexity, using a 0-degree and a 30-degree rigid scope. The operation took 2 hours, and blood loss was 150 mL. The patient was discharged 2 months later without any sequelae [4, 5]. We found no reports of neuroendoscopy performed to relieve ASDH with contusion. The probable reason for this is that there are some difficulties with stopping the bleeding from the contusion and oozing from the brain surface via neuroendoscopy. Due to these hemostatic problems, large craniotomy, which allows better hemostatic control, is usually selected for cases that need decompression from the moment of injury.

From our experience of two cases presented earlier, we would like to recommend the choice of neuroendoscopic surgery on cases of ASDH that are able to be observed clinically without immediate surgery but are expected to gain better outcomes (e.g., efficiency of rehabilitation) through surgical intracranial pressure reduction. For such cases, we also recommend the wait time, if possible, of about 48 hours before surgery for better hemostatic control.

In our cases, it was not our original intent to wait for 48 hours after the traumatic accident. Our initial plan was to give conservative treatment, but since the patients' consciousness level gradually deteriorated, we decided to switch to surgical treatment. As for the method of surgery, neuroendoscopy was chosen because the patients were elderly with multiple complications. During the surgery, we did not experience any difficulty in hemostasis; this is why we considered that 48 hours of wait time may have brought a natural hemostasis and thus resulted in a safer endoscopic surgery. However, it goes without saying that continuous assessment of consciousness and frequent follow-up CT examinations are required during the wait time. Surgical treatment should immediately be applied to patients when deterioration of consciousness is observed. For our cases, we also did a careful checkup of coagulation factors during the wait time because the use of tranexamic acid may slightly increase the risk of thromboembolic events.

Endoscopic surgery performed under local anesthesia is much less invasive compared to the traditional surgery, resulting in a faster postoperative recovery. As for our two patients, their conditions improved soon after the operation and both followed a good postoperative course. Thus, we consider that the 48–72 hours of wait time did not affect their clinical outcome. The reason for the increased hospital stay in our cases was that the patients lived alone with no family and therefore took longer time to be transferred to a rehabilitation hospital. Although the hematoma removal was incomplete for both cases, we consider that this was not related to the increased hospital stay. Total removal of hematoma is considered unnecessary if partial removal of hematoma is sufficient enough to alleviate the mass effect because the remaining hematoma gets absorbed naturally. Even in large craniotomy, there are cases when we leave some hematomas untouched, especially ones that are located around the skull base and the bridging vein.

There may be some concerns over the removal of contusion, since the contusion is normally reserved in order to improve functional outcomes. However, when a contusion is so massive that it forms an intracerebral hematoma over 30 cc and exerts a mass effect, removal of contusion (=intracerebral hematoma) needs to be considered. For such cases, a simple decompressive surgery may not be sufficient to decrease the intracranial pressure, and thus removal of intracerebral hematoma may be required. As for the method of surgery, we often have no choice but to perform craniotomy for cases of massive contusion in the temporal lobe, because those contusions produce early brainstem compression. On the other hand, for massive contusions in the frontal lobe, we are often able to take a wait-and-see approach, so these are possible candidates for neuroendoscopic surgery. In our case, the patient had an intracerebral hematoma caused by a massive contusion in the right frontal lobe. We planned to control the intracranial pressure by removing the massive intracerebral hematoma together with the subdural hematoma under endoscopic surgery.

Last but not least, we would like to point out that although we are currently unable to perform neuroendoscopic surgery at an acute stage due to the difficulty of hemostasis, it may become possible in the future in response to the development of endoscopic hemostatic devices.

### 3.2. Technical Methodology

We make 1 burr hole in the direction of the long axis of the ASDH. We do not make it on the convexity, because that location imposes a limitation for neuroendoscopy. We locate the burr hole in front of the contusion if the patient has massive contusion with ASDH. We can remove both ASDH and the contusion by doing it this way.

When we remove the hematoma by neuroendoscopy through the forehead, it is easy to remove the contusion, but it is important to guide the Neuroport to the subdural space in a skillful manner. Firstly, we guide the Neuroport into the subdural space after removing the contusion omnidirectionally. Then, we move the Neuroport to the outside and continuously feed it into the subdural space beyond the contusion. After that, we can advance the Neuroport for about 6 cm. Gradually, we manage to recognize the Sylvian vein. Further aspiration would lead to bleeding, so we suggest not advancing further after recognition of the Sylvian vein.

This is technical advice, but deep lying hematoma in the brain can easily be suctioned, since there are very few vessels in the deep matter. However, vessels are rich in the subpial space, and frequent electrocoagulation using suction coagulation device is necessary. In addition, we do not recommend the use of a flexible scope, because its suction effect is somewhat lacking. We recommend using a suction instrument to reduce the hematoma through a rigid scope. A flexible scope can cause impairment of the brain directly. In contrast, we can use the Neuroport attached to the rigid scope as the brain retractor.

For safety, we recommend the placement of an information drain into the subdural space in order to check the postoperative bleeding, because we cannot stop bleeding completely insomuch as does a large craniotomy.

In terms of cosmesis of Case 2, it would have been better for the incision to be placed in the hairline like Case 1. However, Case 2 was an exceptional case in which a massive intracerebral hematoma on the right frontal lobe exerted a mass effect on the brain. There was a necessity to make the skin incision on the middle of the forehead in order to remove the intracerebral hematoma together with the subdural hematoma. We placed a 4 cm incision parallel to the wrinkle line and used 5-0 nylon suture for skin closure. In this way, we managed to make the scar hardly noticeable after suture removal. In case of a complication, additional scar would have been required in order to perform craniotomy in Case 2, but it should be noted that this was an exceptional case due to the location of hematoma. If the incision can be placed on the hairline as in Case 1, it is possible to connect the incision with a regular craniotomy incision.

## 4. Conclusion

For patients with underlying medical issues or other risk factors, craniotomy could be unbearably invasive. For those patients, after hemostasis is complete, neuroendoscopic removal of the hematoma and brain contusion is likely to be an effective emergency procedure.
